# Adaptation in landlocked Atlantic salmon links genetics in wild and farmed salmon to smoltification

**DOI:** 10.1186/s12863-024-01263-5

**Published:** 2024-08-30

**Authors:** Cairnduff R, Kjærner-Semb E, Ayllon F, Solberg M, Andersson E, Primmer C.R., Perry W, Glover K.A, Wargelius A

**Affiliations:** 1 Institute of Marine Research, Bergen, Norway; 2 Faculty of Biological and Environmental Sciences, University of Helsinki, Helsinki, Finland; 3 Institute of Biotechnology, Helsinki Institute of Life Sciences (hiLIFE), University of Helsinki, Helsinki, Finland; 4 Cardiff University, Cardiff, UK

**Keywords:** Smoltification, Landlocked, Anadromous, GTSeq, GWAS

## Abstract

Increased knowledge of heritable traits in Atlantic Salmon (*Salmo salar*) is important to overcome bottlenecks in salmonid aquaculture. Atlantic salmonid populations, both landlocked and anadromous, represent an interesting model to gain insight into anadromy related traits, most notably, the probability to smoltify. While a previous study has identified several genomic regions diverging between anadromous and landlocked populations across the species range, the present study explores these data further with the aim to uncover if some of these genomic regions are linked to beneficial genetic traits associated with smoltification. In this study 17 of these loci were monitored in 669 anadromous salmon originating from 36 full-sibling families that had been reared under common garden conditions. The Smolt Index was calculated, using multiple visual markers, and provided a means of assessing smoltification stage. One SNP, located in *Ssa04*, showed a significant association with probability to smoltify, where individuals homozygous for the landlocked variant (LL) displayed a decrease in probability of smoltifying after one winter when compared with the homozygous for the anadromous variant (AA). This effect was independent of individual fish size. A separate common garden study comprising 200 individuals from either anadromous or landlocked strains showed that expression levels of *ncor1,* a thyroid mediator hormone located on the same chromosomal region (*Ssa04),* were significantly reduced in landlocked individuals post smoltification but remained constant in their anadromous counterparts. This study therefore suggests that while size is still the most important trigger for the induction of smoltification, there may also be an additional genetic component or trigger that has been ‘lost’ during the years deprived of SW transfer. In conclusion, the LL genotype identified here could potentially be used by the industry to delay smoltification and may also represent one of the first clues to the genetic regulation of smoltification in Atlantic salmon.

**Supplementary Information:**

The online version contains supplementary material available at 10.1186/s12863-024-01263-5.

## Background

Atlantic salmon (*Salmo salar*, Linnaeus 1758) aquaculture plays a crucial role in Norway's commercial exports, and it is anticipated that the annual production could reach 2.5-3million tons by 2050 [1]. However, the industry currently faces significant challenges, particularly during the transition from freshwater (FW) to seawater (SW) known as smoltification [2]. The production bottlenecks, experienced during this stage of the aquaculture rearing, are primarily attributed to the industry's approach of producing large, rapidly growing smolts to enable year-round production; smoltification is a critical phase of the salmon's life cycle and can result in various complications such as increased levels of mortality and disease in the sea. Consequently, approximately 10% of the mortality occurring in sea cages can be directly attributed to individuals not fully smoltifying, leading to suboptimal harvesting quality [3]. This not only presents significant economic implications but also raises ethical concerns regarding the salmon welfare, impacting the industry’s future development.

Previous studies have described various endocrine-driven physiological changes that occur during the transfer to seawater [4]. These variations encompass changes in morphology [5, 6], immunology [7], and osmoregulatory capacity [8]. Such life history transitions are triggered by photoperiod and temperature and are mediated by hormones including growth hormone (GH), insulin-like growth factor I (IGF-I), thyroid hormones (THs; thyroxine [T4] and 3,3′,5-triodo-l-thyronine [T3]), and cortisol [9]. It is known that salmon parr must reach a certain size threshold, typically between 10-12 cm in length, to undergo smoltification [10]. When parr surpass this threshold, the increase in temperature and day length during the spring triggers smoltification [11]. While the influence of these external cues has long been shown in salmonids, understanding of the underlying genetic pathways have proven relatively elusive. However, the key to understanding these traits may lie within the inherent genetic plasticity in these populations. Heritability of smoltification traits has long been studied amongst salmonids, including rainbow trout (*Oncorhynchus mykiss*) and coho salmon (*Oncorhynchus kisutch)* [12–16]. Previous work has adopted whole genome approaches to compare varying genetic populations of *O.mykiss,* highlighting alleles and genes under direct selection in landlocked strains, suggesting a confluence of genetic markers associated with migration [17]. Additionally, specific chromosomal regions have been identified, with the influence of potential candidate genes being suggested [18]*.*While previous studies have shown phenotypic variation between landlocked and anadromous individuals in Atlantic Salmon, [19, 20], studies investigating the link between the genetic variation and the expressed smoltification traits have been limited.

Previous studies in rainbow trout have shown that multiple QTL’s influence the propensity for displaying smoltification traits in salmonids. Previous studies have shown several multi loci regions including QTLs associated with growth, condition factor, morphology and osmoregulatory enzymes [12, 13].However, the influence of genetic background and heritability of smoltification traits, remains largely unknown in Atlantic salmon.

One potential avenue for identifying genetic variation underlying smoltification traits is in examining and comparing genomes of anadromous and landlocked salmon populations, each inhabiting systems with different salinities. Since the end of the previous Ice Age approximately 10,000 years ago, multiple salmon populations across the Northern Hemisphere have become landlocked, resulting in their exclusion from the seawater migration [21]. Following the retreat of the glaciers and subsequent environmental changes, these landlocked populations underwent adaptation to freshwater environments with likely selection against, or relaxation of selection for, traits associated with smoltification. Consequently, this situation presents an opportunity to identify and compare genomic regions associated with smoltification that are under selection in either landlocked or anadromous populations of Atlantic salmon. By comparing twelve anadromous and six landlocked salmon populations across the Northern Hemisphere we have previously identified 28 genomic regions experiencing distinct selection pressures [22]. However, the functional properties of these genomic regions remain unknown. It could therefore be hypothesized, that some of the genes under selection in these regions could prove crucial for the ability to smoltify. As a result, elucidation of their role in these mechanisms could provide a key insight into improving the smoltification character of industry smolts.

The main goal of this study is to examine whether any of the genomic regions that show signals of selection between landlocked and anadromous Atlantic salmon are associated with smoltification traits in anadromous salmon. To achieve this, we conducted two complimentary experiments 1: a combination of pedigree-based genotyping, which involved analyzing genetic information from individuals with varying smoltification phenotypes, and 2: analyzing expression levels of genes at relevant loci, in a smoltification trial encompassing both landlocked and anadromous strains of salmon.

## Methods

### Smoltification phenotype (experiment 1)

**Fig. 1:**
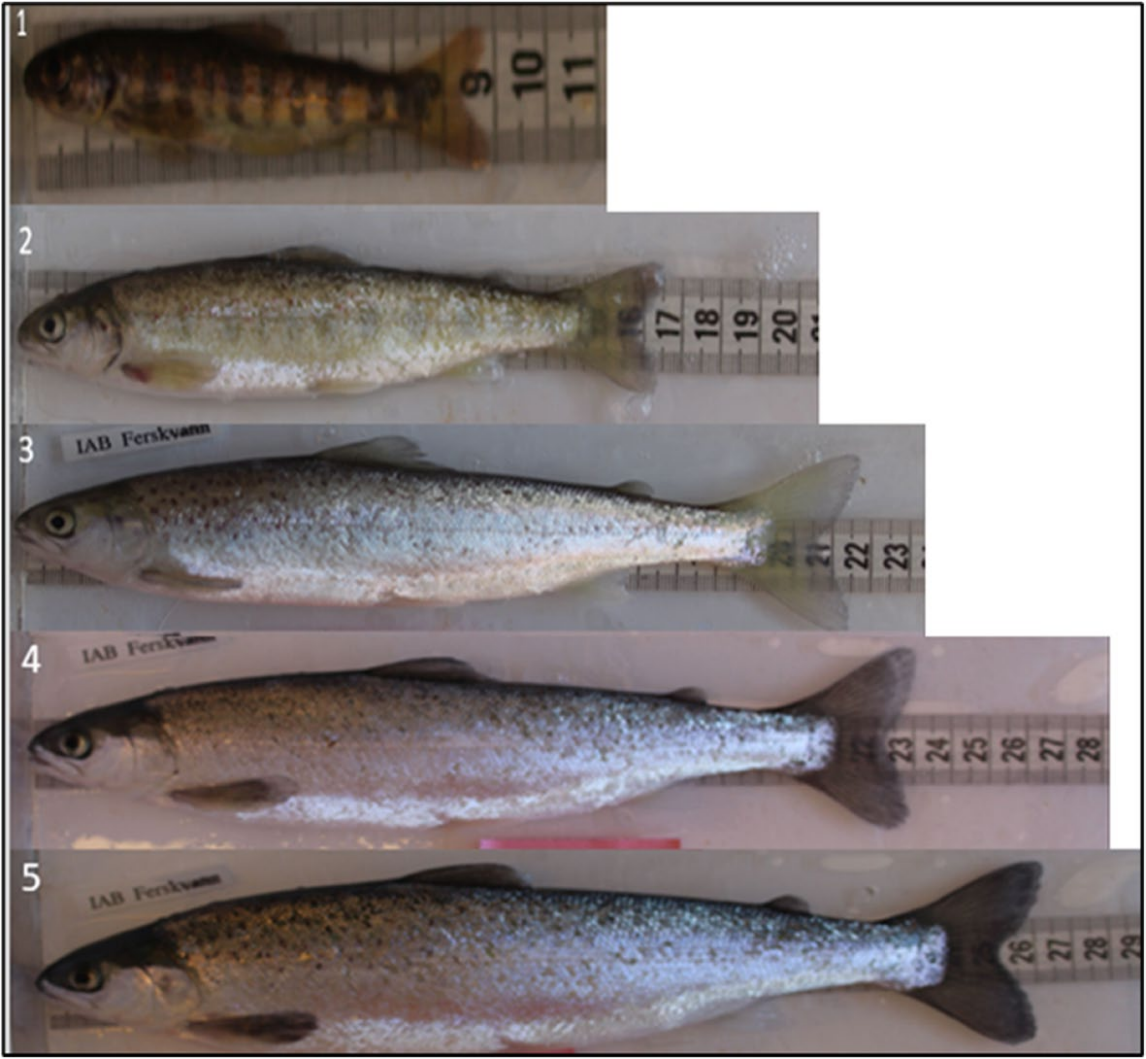
Guide used for visual representation of Smoltification characteristics and scoring. Parr/Non-smoltified fish represented pictures 1 & 2, and smoltified fish represented by pictures 3–5

The experiment consisted of 669 one-year-old Atlantic salmon smolts originating from thirty-six families of anadromous salmon raised under controlled conditions in a common garden setting, with all the fish kept within the same tank, under the same conditions, for the duration of the experiment. These families were derived from a diverse range of naturally anadromous individuals of pure wild (*n* = 6 families), pure domesticated (farmed Mowi) (*n* = 6 families), and various hybrid families (*n* = 24), with hybrid families displaying varying levels of domesticated genome admixture ranging from 25 to 75%: (Domesticated BC (6 Farmed Mowi x hybrid crosses), F2 (6 F1 (Farmed x Wild Hybrid) x F1 crosses,), HWF (6 Farmed mother x wild father crosses,), HFF (6 Farmed father x wild mother,), Wild BC (6 Wild Figgjo x hybrid crosses,) (For detailed information of the experimental conditions and genetic background of the experimental fish, see Perry et al., [23]). Throughout the study, morphometric data, such as photographs, length, and weight, were collected from 669 spring smolts in freshwater (FW) under natural photoperiod and temperature for their natal region during that time of the year. Additionally, fin clips were taken, preserved in ethanol, and used sex the fish using a qPCR assay of the sex determining gene on the Y-chromosome (sdY), described in Ayllon et al., 2020. Measurements and photographs were taken for each fish between 25.04.20 and 05.05.20. Photographs were captured from the lateral side using a digital single lens reflex camera (Olympus TG-860) mounted on a measurement board, with a scale in shot and under natural light conditions. All photographs were also quality checked, prior to any classification of smoltification and low-quality images were removed. The degree of smoltification was determined through a double-blind analysis of the photographs, hence two scientists in the group evaluated the degree of smoltification independent of each other. This analysis involved assigning a classification key based on existing literature [24]. We evaluated the smolt scores by employing three criteria: the presence of parr marks, the extent of silvering, and the coloring of fin margins. Each criterion was assessed on a scale ranging from 1 to 5 (Fig. 1). To conduct the analysis, we grouped the samples into two distinct categories. Fish with scores ranging from 1 to 2 were categorized as non-smoltified individuals (NS), while fish with scores ranging from 3 to 5 were classified as the smoltified subgroup (S).

### Genotyping-by-sequencing (GT-seq)

To genotype SNPs representing the 28 genomic regions identified by the Genome Wide Association Study (GWAS) conducted by Kjærner-Semb., we used Genotyping-in thousands by sequencing (GT- Seq approach [25] We assessed these 28 regions and found that 17 had suitably few repeating sequences, allowing the amplification of only one product from appropriate primer sets for GT-seq analysis. For these regions we looked at the difference in average allele frequency between the landlocked and anadromous populations that have previously highlighted in Kjærner-Semb 2022*.*, Sequence complexity was also into account for designing locus-specific primers suitable for multiplexed PCR. Due to the highly repetitive nature of some of these regions, it was only possible to design primers for 17 of the selected regions. The primers were designed using Geneious Prime (Dotmatics, MA, USA) with the following criteria: primers were required to be 19–21 bp, with optimal GC-content of 55% with added 5’ tails for attaching sequencing adapters. Optimal amplicon size was set to 160 bp. Primer sequences for each locus are included in Supplementary File 1, SX1.

Genomic DNA used for GT-seq analysis was extracted from alcohol-preserved fin-clip samples obtained from both the parents and offspring. The HotSHOT method was employed for DNA extraction [26]. In all samples, genomic target regions were amplified using Qiagen Multiplex PCR Master Mix (Qiagen), followed by a second PCR using Q5® Hot Start High-Fidelity DNA Polymerase (NEB) to generate amplicons containing sample-specific dual-indexed adapters (Supplementary File, SX2) for MiSeq (Illumina). Equal volumes of PCR products were pooled and the QIAquick Gel Extraction Kit (Qiagen) was used to extract the gel bands corresponding to indexed amplicons. Pooled amplicons were sequenced using MiSeq kit v.3 (Illumina) with 300 bp paired end reads. Samples were demultiplexed based on adapter indexes, and forward and reverse reads were assembled to improve sequence accuracy.

Sequence reads from each sample were mapped to the Atlantic salmon reference genome (ICSASG_v2) using Bowtie2 (v. 2.3.5.1, PMID:22,388,286). Samtools mpileup (v.1.10, PMID:21,903,627) was used to obtain allele counts for each locus per sample. Genotypes were called based on read depth and frequency of sequenced alleles at a given locus with the following requirements: Read depth at a locus was required to be at least 50 reads per individual sample. For a sample to be assigned a homozygous genotype, the frequency of reads supporting a given allele had to be > 90%. Heterozygous genotypes were assigned if the frequencies of reads supporting both alleles were within 25–75%.

### Association between smoltification and genetic variants

To explore the impact of these loci on the probability of smoltification during the first year we approximated the posterior distribution of the tested variables using a Bayesian MCMC (Markov Chain Monte Carlo) approach. A univariate mixed-effect model assuming a Bernoulli distribution (probit link) was fitted to assess the effects on the probability of smoltification of several fixed factors: genotypes at a specific locus (*G*), weight at smoltification (*W*), and sex (*S*), as well as all possible two-way interactions between these factors. Additionally, an inverted genetic relationship matrix was fitted to account for kindship as random effect (*a*). A fixed residual variance of 1 and a χ^2^ prior distribution for the additive genetic variance were used to define the priors to assure proper convergence and autocorrelation (suppl. file 1). The response variable, representing the probability of smoltification, was coded as a binary trait with values of 0 (indicating no smoltification) and 1 (indicating smoltification), based on the smoltification index values. Thus, the probability of smoltifying at a given size and sex was fitted using MCMCglmm [27] as follows:

$$Y\text{i }\sim \text{ B}(probit-1(li))$$

$$li \sim \alpha +\beta 1*\text{G}i+\beta 2*\text{W}i+\beta 3*\text{S}i+\beta 4*\text{G}i*\text{W}i+\beta 5*\text{G}i*\text{S}i +\beta 6*\text{W}i*\text{S}i +ai+ei$$

where *Y* is the probability of smoltification of the *i* individual, *B* is a draw from a Bernoulli distribution, and* I* is the liability on the probit scale of the individual *i*. A vector or breeding values (a), including the additive genetic variance and the additive genetic relatedness matrix (*a*), as well as residuals values (*e*) were also included. Model selection was performed backwards by means of the Deviance information criteria approach (DIC). By this procedure, insignificant fixed effects were eliminated, and models were refitted dropping single terms until no further improvement could be detected [28]. Interaction terms were removed before the variables themselves (if significant two-way interaction terms were detected both variables were included in the final model, regardless of their significance level). All statistical modeling was performed using R version 4.2.1 (R Core Team, 2021) and the output of this is included in Supplementary File 2.

### Smoltification trial (experiment 2)

To establish a connection between genetic variants and gene expression levels, an in vivo pilot study was conducted. The objective of this experiment was to examine and compare the gene expression patterns of specific genes located in genomic regions that differ between landlocked and anadromous strains of salmon. The investigation focused on assessing gene expression levels before and after smoltification, with the primary goal of determining whether any differences existed in gene expression within these regions between landlocked and anadromous strains of salmon.

#### Rearing and sampling (or experimental setup)

The fish used in this study were all reared and sampled at the Institute of Marine Research station at Matre (Matredal, Norway) and comprised of individuals from a landlocked population from Gullspång (Sweden) and an anadromous Norwegian farmed (Mowi) strain(Norwegian Animal Research Authority, NARA, permit number 4268). Gullspång individuals had previously been kept for two generations at the facility, and the Mowi for one generation from a previous experiment [18] both were under conditions like standard commercial fish farming. From the embryo stage they were kept in separate tanks under ambient conditions for 1 year. On 16.1.2020, prior to the first sampling, 102 fish were sedated (70 mg/L Finquel), measured for length and weight (~ 30.1 g), fin clipped (adipose fin) and PIT (passive integrated transponder) tagged. The fin clips were stored in ethanol at -20C until DNA was extracted using the Qiagen DNeasy®96 Blood & Tissue Kit (Qiagen). This material was used as a control for testing the GT-seq panel. Welfare and use of these experimental animals was performed in strict accordance with the Norwegian Animal Welfare Act of 19th of June 2009, in force from 1st of January 2010. All personnel involved in the experiment had undergone training approved by the Norwegian Food Safety Authority. This training is mandatory for all personnel running experiments involving animals included in the Animal Welfare Act.

The fish were then placed back into a common garden tank and were reared under ambient conditions, before being lethally sampled at 2 key time points (1st 20.2.20, 2nd 20.5.2020,), bookending the natural spring smoltification at + 1-year smoltification. The visual markers for smoltification were also determined by an experienced handler and the smoltification status and Condition Factor (CF) noted in each case. For each lethal sampling we recorded length and weight of 10 Gullspång and 10 Mowi fish and tissue samples of the gill, head kidney and muscle from the flank were collected and stored in RNAlater at -20C. Four lethal sampling points were planned at one-month intervals, however, this sampling plan was disrupted due to the onset of the 2020 Covid-19 pandemic and localized quarantine measures at the facility and thus only the initial sampling on 20.2.2020 and the final sampling in 20.5.2020 were possible.

#### RNA extraction and qPCR on candidate genes

Candidate genes for this study were selected based on their location in differentiated regions found in the GWAS conducted by Kjærner-Semb et al. [22]. While the majority of these genes had not been looked at in a Smoltification context prior, their physiological characteristics in other model organisms lead us to postulate their perceived differential expression during seawater transfer, in physiologically relevant tissues (gill, liver, head kidney, muscle, and brain). Genes were divided into functional categories (osmoregulation (smoltification), fatty acid metabolism and disease resistance) based on annotations in UniProt (www.uniport.org) and available literature. Primers for qPCR were designed using BatchPrimer3 (http://wheat.pw.usda.gov/demos/BatchPrimer3/) for 15 shortlisted candidates. Primer efficiencies were calculated for each target tissue, using a 4 step dilution series. Standard curves were made for each gene/assay to confirm linear amplification. Primer sequences and efficiencies are listed in Supplementary File 1, SX3.

Total RNA from the tissues collected in the smoltification trial were isolated using Maxwell ® HT simplyRNA Kit on a Biomek 4000 pipetting robot, according to the manufacturers’ instructions. RNA concentration and purity was determined using NanoDrop NP-1000 spectrophotometer (NanoDrop technologies, Wilmington, DE, USA). cDNA was synthesized with SuperScript VILO cDNA Synthesis Kit (Invitrogen), using 1 μg normalized RNA, following the protocol of the manufacturer. qPCR reactions were set up in triplicate with *elongation factor 1 alpha 1* (*ef1a*) as a reference gene. Each reaction mix per sample included: 2 µl FW and RV primers at 5 mM, 3 µl Fast SYBR Green Master Mix (Applied Biosystems. The relative gene expressions were calculated by applying the method of Comparative Ct (or 2^−ΔΔCt^) [29]. The data was calibrated to the sample with the lowest Ct value. No gene expression variation was found in between fish strains for the reference gene.

#### Statistical Analysis of gene expression

We conducted statistical analyses using GraphPad Prism 9.4.1 (GraphPad Software, Inc.). First, we assessed the normal distribution of all datasets using the Shapiro- Wilk normality test. Technical replicates for each of the samples were ran in triplicate, as is standard for ∆∆Ct and a one-way ANOVA was run to assess the variation in results, with any variation greater than 2 standard deviations (95%) being removed. For results deemed to have passed the normality test, a standard two tailed t test was first used to compare between the strains for each time point and then between the two-sampling time points themselves. For datasets failing the normality test or with insufficient sample size (*n* < 8) for normality testing, we utilized the nonparametric 2-sided Mann–Whitney U/ Wilcoxon test.

## Results

### GTSeq analysis of fish from experiment 1

The alleles typically found in landlocked populations (L) were found to be fixed in the landlocked Gullspång population across 14 of the 17 loci (Table 1). In contrast, these alleles were found at low frequency in the anadromous domesticated strains. In these strains, anadromous alleles (A) were close to fixation for 8 out of 17 studied SNPs, while the remaining 8 loci were more heterogeneous, with a mix of both A and L alleles. Also, the three loci on Chr 24, allele frequencies were found to be evenly distributed, with the L allele frequencies of 0.71, 0.42 and 0.55 for SNPs Chr24:17,705,276, Chr24:18,531,930 and Chr24:40,053,680, respectively (Table 2).

**Table 1:**
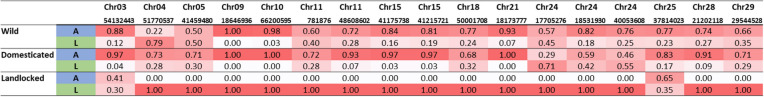
Frequencies of alleles across 36 families of *Salmo salar; *wild(6 Figgio Wild strain), farmed (6 Domesticated Mowi) and landlocked Gullspång fish study (n) across 17 SNPs utilized in a GTSeq panel. The degree of red coloring highlights the major allele frequency. A= Anadromous Allele, L= Landlocked Allele

**Table 2:**
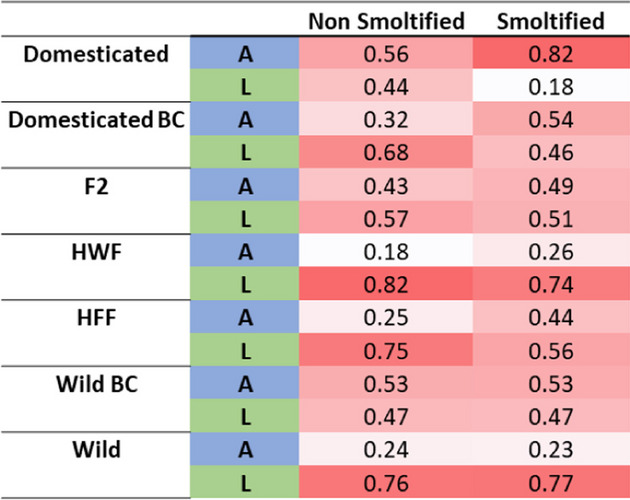
Average frequencies of alleles across smoltified and non-smoltified individuals of *Salmo salar, *broken down into different strains*; *Domesticated(6 Farmed Mowi families, *n*=91), Domesticated BC (6 Farmed Mowi x hybrid crosses, *n*=90), F2 (6 F1 (Farmed x Wild Hybrid) x F1 crosses, *n*=91), HWF (6 Farmed mother x wild father crosses, *n*=57), HFF (6 Farmed father x wild mother, *n*=42), Wild BC  (6 Wild Figgjo x hybrid crosses, *n*=117) & Wild (6 Figgio families, *n*=81) across 17 loci examined by the GTSeq panel. The degree of red coloring highlights the major allele frequency. A= Anadromous Allele, L= Landlocked Allele

The anadromous wild strains showed more genetic variation among the assayed alleles compared to the landlocked strains, with only one single SNP being fixed for the A allele (Chr9:18,646,936), with 3 other SNPs also displaying the A allele at a high frequency in the anadromous families (Chr3:54,132,443, 0.88; Chr10:66,200,595, 0.98; Chr21:18,173,777, 0.93, see Table 1). Eleven of the other regions showed an even distribution between the A and L alleles, with Ssa04:51,770,537 being the only region to display a higher frequency of the L allele (0.79).

### Probability of smoltification

**Fig. 2:**
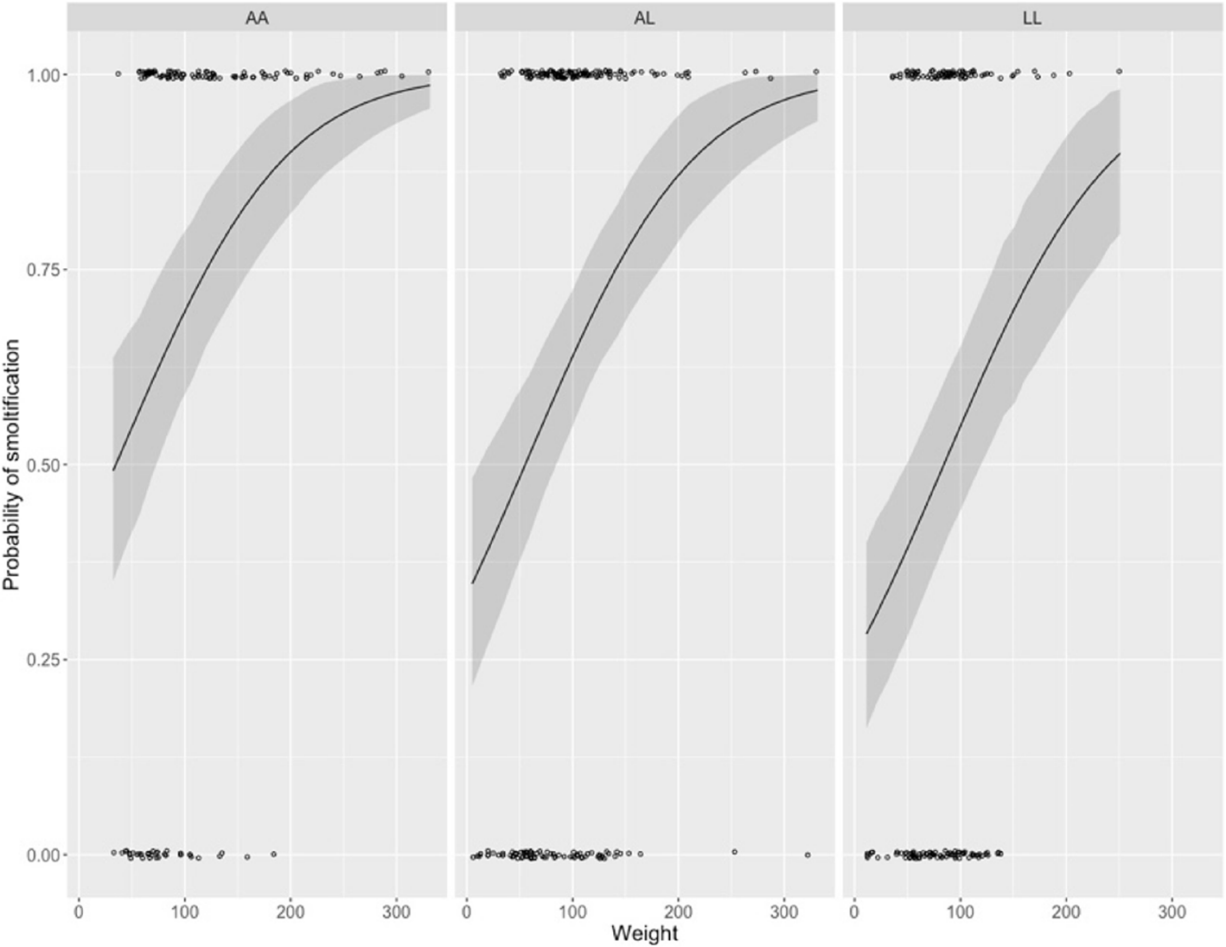
A series of line plots detailing the probability of smoltification of 1 year old Atlantic Salmon (*Salmo salar*). Solid lines represent the predicted probability of smoltification (y-axis 0–1) as a function of weight at smoltification (x-axis 0-350 g) for the observed genotypes (*AA, AL,* and *LL*) at the Ssa04:51,770,537 locus. Shadow areas represent 95% confidence intervals for the predicted probability. Data points are shown as black circles

Among the studied variables and their two ways interactions, sex and all possible two ways interactions were found to be significantly penalizing model fit (based on DIC values) and were removed. Consequently, we favored a model including weight at smoltification and one SNP (Ssa04:51,770,537) and found these variables to be associated with the probability of smoltification (Fig. 2). Thus, individuals showed strikingly higher odds of smoltification as the final weight increases. On the other hand, the presence of the L allele on Ssa04 (Table 0.1) was shown to decrease the probability of smoltification regardless the weight at smoltification. Thus, heterozygous individuals (*AL*) showed a lower probability of smoltification than individuals homozygous for the anadromous allele (*AA*). Furthermore, homozygous individuals for the landlocked variant (*LL*) showed a clear trend for a reduced probability of smoltification regardless of size, especially when compared with their homozygous *AA* counterparts (Fig. 2).

### Expression of candidate genes in landlocked and anadromous salmon before and after smoltification (experiment 2)

**Fig. 3:**
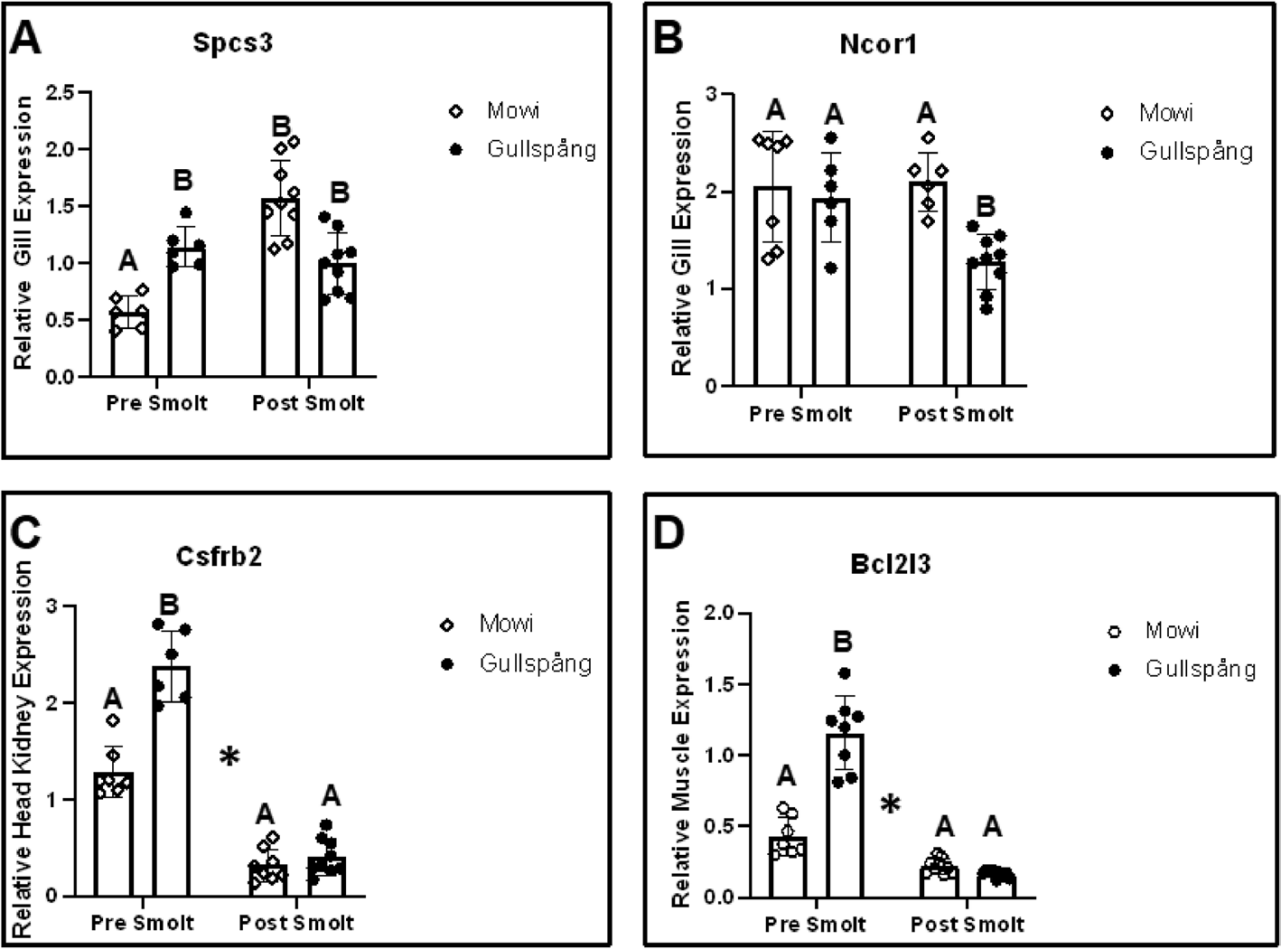
Bar Graphs showing relative gene expression of several key candidate genes potentially associated with smoltification in Gill (A, *spcs3*,B *ncor1*) head kidney (C *csfrb2)* and muscle (D, *bcl2i3*); before (Pre) and after smoltification (Post) in 1 + year old Atlantic Salmon (*Salmo salar*) individuals. Significant differences in gene expression, between strains at each time point are denoted with different letters while differences between time points are demarcated with a star* (*P* < 0.05)

Fifteen candidate genes previously linked to osmoregulation (specifically, smoltification) were selected from the genomic regions previously identified as under selection by comparing the genomes of landlocked and anadromous salmon (Kjærner-Sem et al., 2002). Gene expression analysis was performed by qPCR on gill, head kidney and muscle in farmed anadromous (Mowi) and landlocked (Gullspång) salmon before and after smoltification (Fig. 3). Among the 15 examined genes, a total of four genes (*ncor1*, *spcs3*, *csfr2b2*, *bcl2l3*) exhibited differential expressions across the two timepoints within each strain (Fig. 3). Additionally, three of these (*spcs3*, *csfr2b2*, *bcl2l3*) also demonstrated differential expression between the strains at across the timepoints.

Relative expression of *ncor1* increased significantly in gills from Mowi fish after smoltification, while a significant decrease was observed in the Gullspång fish, while no significant difference was noted in the other tissues assayed.

Relative expression of *spcs3* was differentially expressed in both the gill and HK. In both tissues no significant increase was noted in the Gullspång fish before and after smoltification, but a significant increase was noted in the Mowi strain.

Relative expression of *csf2rb2* was differentially expressed in the head kidney. No significant change was noticed in the Mowi individuals across the timepoints, but levels significantly reduced between the time points for the Gullspång. Additionally, levels in the Gullspång were significantly higher in the pre smoltification individuals than the comparative Mowi individuals at the same timepoint.

In muscle, relative expression of b*cl2l13* was found to be significantly higher in Gullspång compared to Mowi fish prior to smoltification. Both strains displayed a significant reduction after smoltification and were no longer significantly different at this stage.

## Discussion

In this study, we have employed a sequencing based approach to investigate whether genomic regions experiencing selection in landlocked salmon are associated with key genes smoltification process. To achieve this, we utilized a combination of SNP analysis using GT-seq methodology on pedigreed samples with contrasting smoltification phenotypes, along with candidate gene expression analysis of anadromous and landlocked salmon before and after smoltification. This work revealed one locus in *Ssa04*, in which individuals homozygous for landlocked alleles (LL), displayed a significantly lower probability of smoltification at age 1 + that was independent of individual fish weight. While the results clearly show the trend that size is an important factor to smoltification, as supported by previous literature [30, 31], our data suggest that there may be weight independent genetic variability that contributes to smoltification in Atlantic salmon. Also, unknown genotypes of the admixed fish could have affected the smoltification trait.

Unsurprisingly, also in our pedigreed material, the major factor contributing to the probability of smoltification was the weight of the individuals. Salmonids typically spend at least a year in FW to achieve the minimum size required to smoltify, with this occurring for Atlantic salmon at 70-100 g under typical farmed conditions [10]. Interestingly, one of the genomic regions on *Ssa04* was associated with smoltification, independent of weight of the fish. It further indicates that homozygous LL would need to reach larger sizes than the AA fish to smoltify. This suggests that this region, particularly the genes contained within it, are likely to be influential to smoltification. The link between this region and smoltification has never previously been reported. A previous study using a quantitative genetics methodology, found no association between genetic variation and smoltification status [24].

The pedigreed material used in this study consisted of wild, hybrid, and domesticated fish. Notably, the prevalence of the L allele is less common in the farmed strain (0.28) compared to wild strain (0.76). This discrepancy suggests that unintentional selection during the breeding of farmed salmon over the past four decades may have favored the A allele, resulting in the inadvertent reduction in the L allele frequency in farmed strains. In the early stages of the Atlantic salmon industry, some juvenile fish (parr) would undergo partial smoltification in spring, approximately 16 months after hatching. Those that did not fully smoltify at this stage, known as (1 +) smolts, would continue to be reared in freshwater. Only after another year, around 28 months after hatching, would the remaining parr undergo complete smoltification, becoming (2 +) smolts [30]. In modern domesticated Atlantic salmon production, the production of smolts is limited to those production schemes below 16 months of age, commonly referred to as (1 +) or (0 +) smolts. The production of (2 +) smolts does not occur in industry, and maybe this production feature, and not the targeted breeding schemes, has resulted in selection against the L allele. However, the reintroduction of the *Ssa04* L allele into farmed strains could potentially be beneficial for producing larger smolts which is currently of interest to the industry. Notably, the L allele appears to reduce the probability of smoltification across the weight distribution of our study, adding to its appeal. However, to assess the potential benefits of utilizing this allele, further studies using pedigree material focused on large smolt production and subsequent performance in seawater environments need to be conducted. Additionally, it is of interest to investigate the functional properties of this genomic region to potentially identify the causal variants responsible for delayed smoltification.

The 200 kb genomic region of *Ssa04* [51,, 750,, 000–51950,, 000], which was represented by 51,770,537 SNP includes 8 annotated genes (*shpk, ncor1, ubi-p63e, p2rx5, emc6, trpv1, pigl, trpv1*) and one uncharacterized gene [18]. While any gene in that region could be potentially linked to the observed phenotype, interestingly, *ncor1*, encodes a thyroid transcriptional repressor that possesses functions that directly relate to smoltification. Thyroid hormones have important functions before, during, and after smoltification in salmon, while prior to smoltification, they contribute to fry development and organ maturation [32]. During smoltification, thyroid hormones, especially T3, drive morphological changes, such as coloration and gill remodeling, enabling adaptation to the marine environment. After smoltification, thyroid hormone levels decrease, but they continue to maintain osmoregulatory functions necessary for marine survival. The thyroid has long been understood to play a key role within smoltification [33] with elevated levels of both T3 and T4 hormones found to have increased during the smoltification window, particularly after SW transfer [34]. Thyroid hormones are known to stimulate a variety of changes during the smoltification process, specifically metabolic and morphological changes [35]. Supplementary exogenous thyroid stimulation has been shown to increase both silvering, lipolysis, and hemoglobin isoform proliferation during this time [33]. In terms of salinity tolerance, previous studies have shown that dietary and injection treatments with thyroid hormones elicit only a limited impact on salinity tolerance of salmonids [9]. This would suggest that instead thyroid hormones are more likely to operate indirectly through the GH–IGF-I and cortisol axes [36]. Previously exogenous T3 treatment has been shown to increase abundance of gill cortisol receptors and synergizes with GH to increase them even further [37]. Given that IGF-I, when assayed in the same fish, showed no distinct deviation from the anadromous counterpart, it could be postulated that the excitation of the T3/T4 pathway may act as an indirect compensatory mechanism for the pathway. In mice, NCOR1 acts as a nuclear receptor corepressor and a well-recognized transcriptional coregulator and has been shown to have significant impact on Thyroid Hormone sensitivity when knocked out in vivo in mice [38]. In fact, in mice NCOR1 is the principal regulator of TH action. Knockdown studies in zebrafish have not confirmed this but instead revealed a role of this protein in myelopoiesis and subsequent maturation of macrophages and neutrophils, while thyroid signaling functions have not yet been explored [39]. The upregulation of NCOR1 in the farmed fish after the smoltification window, would indicate suppression of thyroid hormones suggesting that smoltification has already been completed. Conversely, the downregulation of *ncor1* in landlocked individuals after smoltification suggests the possibility of a hyperthyroidic response, potentially leading to increased thyroid stimulation. If the L allele is associated with the gene expression regulation and prospective level of the encoded Ncor1 protein, this could result in altered sensitivity to thyroid hormone signaling, possibly explaining the delayed smoltification.

Additionally, genes previously shown within the *Ssa04* region, highlighted in Kjærner-Semb [22], may also go some way to explaining this potentially energy demanding mechanism. *Ubi-p63E* encodes a polyubiquitin precursor belonging to the family of deubiquitinating enzymes (DUBs), which are in turn known to be involved in GH pathway [40]. *Shpk* encodes a carbohydrate kinase crucial for cellular metabolism involved in the phosphorylation of carbohydrates as they enter a cell. While postulated to be involved in the immune response in adaptive pressures in Chinook salmon (*Oncorhynchus tshawytscha*) [41], this energy production could also be useful during smoltification, a highly energy demanding process. This is further substantiated by the presence of *p2rx5*, a gene that belongs to the family of purinoreceptors for ATP. This indicates, as suggested by Velotta et al., [42], that while polygenic selection contributes to adaptation across salinity boundaries, changes in ATPase enzymes may be of particular importance in supporting such transitions. It should also be noted that the landlocked strain had only been bred for two generations in captivity compared to MOWI strain which has been bred for more than 20 generations, this may also contribute to expressional differences.

While the primary focus of the study has involved investigating smoltification traits, markers for disease resistance and fatty acid metabolism have also been highlighted. Three of the candidate genes from chromosomal regions not highlighted by the GT-seq were shown to be differentially expressed. Both Spcs3 and Csf2rb2 are known to be involved within the immune response, in microsomal signaling and the JAK-STAT pathway, respectively. *spcs3* levels increased for the Mowi individuals after smoltification but remained constant for the Gullspång. Conversely, levels of *csf2rb2* were shown to decrease in the same time frame. This could suggest that the adaptive immune responses in freshwater and in sea water differ, which in turn would explain why non-smoltifying landlocked salmon do not respond well to sea water transfer.

## Conclusions

Through examining previously characterized genomic regions that exhibit contrasting selection patterns in anadromous and landlocked Atlantic salmon populations across the Northern Hemisphere, we explored the relationship between these genomic regions and the likelihood of smoltification at a specific body size. Notably, we identified an association between a SNP from the selective sweep region on Chromosome 4 and probability to smoltify. Individuals displaying a landlocked allele (L) in this region of *Ssa04*, showed a clear reduced probability of smoltification at any given size. This region includes *ncor1*, which encodes a thyroid transcriptional repressor potentially important for smoltification in anadromous individuals. Interestingly, we observed opposite regulation of *ncor1* gene expression, with an up-regulation in anadromous fish and a down-regulation in landlocked fish across smoltification. In summary, the LL genotype discovered here might offer the industry a means to assess the probability of individuals to smoltify (and potentially postpone the process),serving as an early indicator of the genetic control of this process in Atlantic salmon.

### Supplementary Information

Supplementary Material 1.

Supplementary Material 2.

## Data Availability

The datasets generated and/or analysed during the current study are available in the NCBI, BioProject ID PRJNA1048809 ( https://www.ncbi.nlm.nih.gov/sra/PRJNA1048809). Any additional data is also available from the Author.
