# Supplementary material for: Adaptation in landlocked Atlantic salmon links genetics in wild and farmed salmon to smoltification
Source: BMC Genom Data. 2024 Aug 30;25:78. doi: 10.1186/s12863-024-01263-5 (PMC11363631; doi:10.1186/s12863-024-01263-5)
Supplement: Supplementary file 2 — Supplementary Material 2. [file 12863_2024_1263_MOESM2_ESM.docx]

# MCMCglmm analysis Fisher *et al.*

We ran a univariate mixed-effects model to calculate the estimates of each factor significantly contributing to the probability of smoltification of Atlantic salmon after one rear in a freshwater controlled environment. The example below corresponds to the curated data set for the NC_027303.1 locus.

### Load libraries

The version and dependencies loaded are detailed here

library(MCMCglmm)
library(plotMCMC)
library(optiSel)

sessionInfo()

R version 4.2.1 (2022-06-23)
Platform: x86_64-apple-darwin17.0 (64-bit)
Running under: macOS Big Sur ... 10.16

Matrix products: default
BLAS: /Library/Frameworks/R.framework/Versions/4.2/Resources/lib/libRblas.0.dylib
LAPACK: /Library/Frameworks/R.framework/Versions/4.2/Resources/lib/libRlapack.dylib

locale:
[1] en_US.UTF-8/en_US.UTF-8/en_US.UTF-8/C/en_US.UTF-8/en_US.UTF-8

attached base packages:
[1] stats graphics grDevices utils datasets methods base

other attached packages:
[1] optiSel_2.0.7 plotMCMC_2.0.1 MCMCglmm_2.35 ape_5.7-1 coda_0.19-4
[6] Matrix_1.5-1

loaded via a namespace (and not attached):
 [1] rgl_0.110.2 Rcpp_1.0.10 lattice_0.20-45
 [4] corpcor_1.6.10 gtools_3.9.4 digest_0.6.31
 [7] foreach_1.5.2 R6_2.5.1 plyr_1.8.8
[10] magic_1.6-1 alabama_2022.4-1 pedigree_1.4.2
[13] evaluate_0.16 gplots_3.1.3 rlang_1.1.1
[16] cubature_2.1.0 rstudioapi_0.14 data.table_1.14.8
[19] kinship2_1.9.6 nloptr_2.0.3 rmarkdown_2.20
[22] stringr_1.5.0 htmlwidgets_1.5.4 HaploSim_1.8.4.2
[25] compiler_4.2.1 numDeriv_2016.8-1.1 xfun_0.37
[28] base64enc_0.1-3 htmltools_0.5.4 tensorA_0.36.2
[31] quadprog_1.5-8 codetools_0.2-18 shapes_1.2.7
[34] reshape_0.8.9 pspline_1.0-19 MASS_7.3-58.1
[37] bitops_1.0-7 grid_4.2.1 nlme_3.1-159
[40] jsonlite_1.8.4 lifecycle_1.0.3 magrittr_2.0.3
[43] KernSmooth_2.23-20 cli_3.6.1 stringi_1.7.12
[46] reshape2_1.4.4 scatterplot3d_0.3-42 doParallel_1.0.17
[49] vctrs_0.6.2 optiSolve_1.0 iterators_1.0.14
[52] tools_4.2.1 glue_1.6.2 purrr_1.0.1
[55] nadiv_2.17.2 abind_1.4-5 parallel_4.2.1
[58] fastmap_1.1.0 yaml_2.3.5 caTools_1.18.2
[61] cccp_0.2-9 minpack.lm_1.2-3 ECOSolveR_0.5.5
[64] knitr_1.40

###

### Load data frames and model object

The following code will load two data frames containing the phenotypic data for 473 individuals and the pedigree information for the whole population containing three columns with unique IDs that correspond to each individual, its dam, and its sire. To avoid running the model again the model object can be loaded into the workspace.

# Load the phenotypic data
NC_027303.1_df<-read.table("NC_027303.1_df.txt", header=T,colClasses =c("factor","factor","integer","factor",c(rep("numeric",4)), "factor" ))

# Load the pedigree file
ped<-read.table("NC_027303.1_Ped.txt",header=T,colClasses =c("factor","character","character"))

# Load the model object to avoid running the full model
load("/Users/fernando/Desktop/Miscellaneous_Projects/Ross_Smoltification/R Analysis for Reviewers/model_NC_027303.1_object.RData")

###

### Create the inverse relatedness matrix

We need to specify the random effect that must be linked to the pedigree but it is much more convenient to fit the inverse relatedness matrix in to model. We thus first create the genetic relatioship matrix and then estimate the inverse relatedness matrix as follows.

pedX<-prePed(ped)
InvPedX <- inverseA(pedX[,1:3])$Ainv

###

### Prior specification

Due to the impossibility of estimate the additive genetic variance and the residual variance at the same time for binary data, we fix the residual variance (R) to 1 and use a 𝒳^2^ prior distribution for the additive genetic variance (G).

prior <- list(R = list (V = 1, fix = 1),
 G = list (G1 = list (V = 1, nu = 1000, alpha.mu = 0, alpha.V = 1)))

###

### Running the model

The probit link is consider as best practice for pedigree based analysis so we specific the paramater *family* as “threshold”. Autocorrelation issues are inherent to binary data so a rather long run i usually needed to assure proper MCMCM chain mixing. Thus, we used a large number of iterations *1e+08* and a generous burning period of *1e+06* with a thinning interval of *1e+04*. To improved convergence we used the *slice* option and to avoid bias towards extreme probability region of the latent variable (which is common in binary models) we used the option *trunc*. As fixed factors we included both standarized weight (*weight.std*) and the genotype for the investigated locus (*NC_027303.1*). The ID variable matching with the the inverse genetic relatedness matrix was included as a random effect (*ID*). Note of caution: Running times can be extremely long depending on the computer.

model_NC_027303.1<-MCMCglmm(Smolt_Score_with_4 ~ Weight.std+NC_027303.1,
 random = ~ ID,
 family = "threshold",
 data = NC_027303.1_df,
 verbose = FALSE,
 nitt = 10E8, thin =10E4, burnin = 10E6,
 ginv = list(ID = InvPedX),
 prior = prior,
 pr=TRUE,slice=T,trunc = T)

summary(model_NC_027303.1)

Iterations = 1000001:99990001
 Thinning interval = 10000
 Sample size = 9900

 DIC: 585.82

 G-structure: ~ID

 post.mean l-95% CI u-95% CI eff.samp
ID 0.216 1.002e-07 0.6152 9900

 R-structure: ~units

 post.mean l-95% CI u-95% CI eff.samp
units 1 1 1 0

 Location effects: Smolt_Score_with_4 ~ Weight.std + NC_027303.1

 post.mean l-95% CI u-95% CI eff.samp pMCMC
(Intercept) 0.557580 0.229498 0.916330 10202 0.00263 **
Weight.std 0.463852 0.276278 0.653127 9258 < 1e-04 ***
NC_027303.1AL -0.176431 -0.555000 0.201710 9900 0.34667
NC_027303.1LL -0.434333 -0.874464 -0.009667 9900 0.03919 *
---
Signif. codes: 0 '***' 0.001 '**' 0.01 '*' 0.05 '.' 0.1 ' ' 1

###

### Model Diagnostics

Fist we assessed the mixing of the different chains for the fixed and random effects. The trace plots show the evolution of the sampling values across the iterations. It should explore the whole range of the posterior distribution without getting stuck. Typically, the model has converged when the plot looks like a fuzzy caterpillar. Values on the *Y* axis should be |β|<10 to be consider problem-free. In our case, the *units* plot refers to the residual variance which we have fixed to 1, so no variance en the chains is expected.

allChains <- as.mcmc(cbind(model_NC_027303.1$Sol,model_NC_027303.1$VCV))
allChains_sub <- allChains[,c(1:4,512:513)]
plotTrace(allChains_sub,axes=TRUE,las=1)


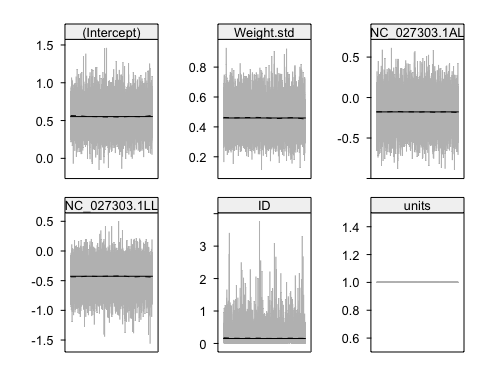


Next we can check the autocorrelation across the chains. Values below 0.1 in Lag >1 are considered acceptable.

cbind(autocorr.diag(model_NC_027303.1$Sol[,1:4]),autocorr.diag(model_NC_027303.1$VCV))

(Intercept) Weight.std NC_027303.1AL NC_027303.1LL ID
Lag 0 1.000000000 1.000000000 1.0000000000 1.0000000000 1.000000000
Lag 10000 -0.015059655 -0.005359764 -0.0126976358 -0.0044714822 0.008995928
Lag 50000 -0.001288383 -0.004205239 0.0006697094 0.0001011059 0.004030205
Lag 1e+05 0.010872966 -0.027945657 0.0017126726 0.0020762408 0.008501747
Lag 5e+05 0.012130364 0.001478797 -0.0016234778 0.0083015600 0.003433457
 units
Lag 0 NaN
Lag 10000 NaN
Lag 50000 NaN
Lag 1e+05 NaN
Lag 5e+05 NaN

We checked the model convergence by means of the Heidelberger and Welch’s MCMC Convergence Diagnostic. It runs length control diagnostic based on a criterion of relative accuracy for the estimate of the mean. It also implements a convergence diagnostic, and removes up to half the chain in order to ensure that the means are estimated from a chain that has converged.

rbind(heidel.diag(model_NC_027303.1$Sol[,1:4]),heidel.diag(model_NC_027303.1$VCV))

stest start pvalue htest mean halfwidth
(Intercept) 1 1 0.2964400 1 0.5575796 0.003399044
Weight.std 1 1 0.1606284 1 0.4638519 0.001970226
NC_027303.1AL 1 1 0.4819700 1 -0.1764313 0.003796377
NC_027303.1LL 1 1 0.3962123 1 -0.4343326 0.004302065
ID 1 1 0.2536304 1 0.2159776 0.004684553
units 0 NA NA NA NA NA

Finally, an estimation of the effective sample size assess that the model retained enough number of independent values. An effective size above 1000 is consider adequate.

effectiveSize(model_NC_027303.1$Sol[,1:4])

(Intercept) Weight.std NC_027303.1AL NC_027303.1LL
 10201.710 9258.171 9900.000 9900.000

effectiveSize(model_NC_027303.1$VCV)

ID units
 9900 0

Estimates of the posterior mode and its confident intervals for each covariate in the model can be obtained

cbind(mode=posterior.mode(model_NC_027303.1$Sol[,1:4]),HPDinterval(model_NC_027303.1$Sol[,1:4]))

mode lower upper
(Intercept) 0.5306571 0.2294982 0.916329652
Weight.std 0.4190948 0.2762778 0.653127290
NC_027303.1AL -0.1544044 -0.5550004 0.201709875
NC_027303.1LL -0.3931858 -0.8744642 -0.009666712

**Acknowledgements**

The authors want to acknowledge the unvaluable online resources regarding MCMC Bayesian Statistics and Animal Models provided by [Jarrod Hadfield](http://cran.nexr.com/web/packages/MCMCglmm/vignettes/CourseNotes.pdf) and [Pierre de Villemereuil](https://devillemereuil.legtux.org/wp-content/uploads/2012/12/tuto_en.pdf).
